# Functional inhibition of heat shock protein 70 by VER‐155008 suppresses pleural mesothelioma cell proliferation via an autophagy mechanism

**DOI:** 10.1111/1759-7714.13784

**Published:** 2020-12-14

**Authors:** Kosuke Sakai, Maya Inoue, Shintaro Mikami, Hiroaki Nishimura, Yoshiki Kuwabara, Akitoshi Kojima, Maiko Toda, Yumiko Ogawa‐Kobayashi, Satoshi Kikuchi, Yusuke Hirata, Yuriko Mikami‐Saito, Hiroyuki Kyoyama, Gaku Moriyama, Michio Shiibashi, Masahiro Seike, Akihiko Gemma, Kazutsugu Uematsu

**Affiliations:** ^1^ Department of Pulmonary Medicine, Saitama Medical Center Saitama Medical University Saitama Japan; ^2^ Department of Pulmonary Medicine and Oncology Graduate School of Medicine, Nippon Medical School Tokyo Japan; ^3^ Information Technology Center Saitama Medical University Saitama Japan

**Keywords:** Gefitinib, heat shock protein 70, macroautophagy, pleural mesothelioma, VER‐155008

## Abstract

**Background:** Pleural mesothelioma, a devastating asbestos‐associated malignancy, urgently requires a novel effective therapy. Heat shock protein 70 (HSP70), which is synthesized in the cell response to protein damage, is expected to be a new target for antitumor treatment. In addition to its well‐known protein refolding function, HSP70 regulates cell proliferation through different pathways, including PI3K/AKT/mTOR, and autophagy in malignant cells. In this study, we attempted to clarify the effects of VER‐155008, an HSP70 inhibitor, on pleural mesothelioma.

**Methods:** Human pleural mesothelioma cell lines 211H, H2452 and H28 were cultured with VER‐155008, and protein expression, cell proliferation, colony formation, cell cycle, synergistic effect with cisplatin, and autophagy induction were analyzed.

**Results:** In mesothelioma cell lines, VER‐155008 (5.0 μM or more) inhibited cell growth and colony formation, accompanied by G_1_ cell cycle arrest. According to western blot analysis, VER‐155008 reduced p‐AKT expression. However, VER‐155008 failed to show a synergistic effect with cisplatin on cell growth. Mesothelioma cells transfected with the novel plasmid pMRX‐IP‐GFP‐LC3‐RFP‐LC3ΔG, which was developed for the quantitative and statistical estimation of macroautophagy, showed enhanced macroautophagy upon treatment with VER‐155008 and gefitinib which is an EGFR‐tyrosine kinase inhibitor. In addition, fetal bovine serum deprivation induced macroautophagy was further enhanced by VER‐155008.

**Conclusions:** On the basis of these results, functional HSP70 inhibition by VER‐155008 suppressed cell growth in pleural mesothelioma cells, accompanied by enhanced macroautophagy. HSP70 inhibition is thus expected to become a new strategy for treating mesothelioma.

**Key points:**

**Significant findings of the study**

In pleural mesothelioma cells, inhibition of HSP70 function by VER‐155008 suppressed cell proliferation accompanied by induction of autophagy which was synergistically enhanced under the starvation condition, whereas gefitinib, an EGFR‐TKI, did not show the same synergistic effect in autophagy.

**What this study adds**

The inhibition of HSP70 induced autophagy and suppressed cell proliferation in mesothelioma cells.

## Graphical abstract

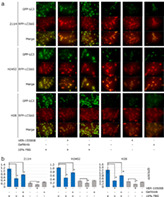

## Introduction

Pleural mesothelioma is attributed primarily to asbestos exposure, and therapies including chemotherapy, radiotherapy and immune checkpoint inhibitors have been adopted clinically; however, its prognosis is still devastating. In Japan, it is estimated that more than 40 000 people will suffer from pleural mesothelioma from 2030 to 2039;^1^ therefore, the development of effective therapies based on the molecular biology of mesothelioma is urgently required.

We have revealed that suppressing disheveled‐3 (Dvl‐3) in the Wnt signaling pathway elicited antitumor effects in mesothelioma cell lines. Furthermore, we found that reduced protein expression of heat shock protein 70 (HSP70) was accompanied by Dvl‐3 suppression in mesothelioma cells by the use of two‐dimensional gel electrophoresis. HSP70 is known to be synthesized in response to increasing temperatures and is expressed in every cell in the body. It is a highly conserved molecular chaperone from eubacteria and archaebacteria to mammals.^2^ Heat shock proteins are also called stress proteins^3^ because they can be induced by any other protein‐damaging factors, such as oxygen‐free radicals and heavy metals.^4^ HSP70 has ATPase activity and refolds intracellular proteins that lack an appropriate three‐dimensional structure through ATP hydrolysis, thereby contributing to the maintenance of cell quality.^5^ In addition to these functions, HSP70 has pleiotropic effects on intracellular signaling pathways. In non‐small cell lung cancer (NSCLC) and colorectal adenocarcinoma cell lines, HSP70 has been demonstrated to activate the AKT/mTOR pathway in the regulation of cell proliferation and G_1_/S phase progression in the cell cycle.^6^, ^7^, ^8^ HSP70 has also been shown to inactivate Bax to mediate stress‐activated apoptosis.^9^, ^10^

In NSCLC cell lines, activation of the AKT/mTOR pathway through HSP70 has been demonstrated to suppress macroautophagy.^8^ Because macroautophagy is also induced by epidermal growth factor receptor (EGFR)‐tyrosine kinase inhibitors (TKIs), which are molecular target drugs for NSCLC patients with sensitive *EGFR* mutations, autophagy is expected to be a new therapeutic target in the treatment of NSCLC independent of EGFR expression.^11^, ^12^ Alternatively, lysosome‐associated membrane protein 2A (LAMP‐2A), a surrogate marker for chaperone‐mediated autophagy (CMA), which is regulated by heat shock cognate 70 (Hsc70), a cofactor of HSP70, has been shown to be increased in NSCLC, breast cancer, hepatocellular carcinoma and osteosarcoma cell lines.^13^ Tumor cells need to obtain the necessary abundant glucose by CMA,^13^ and Hsc70 is the only chaperone that mediates CMA.^14^ Several autophagic mechanisms are known to contribute to supplying the necessary nutrients, particularly for malignant cells, while autophagy induced by reagents does not always act cytoprotectively; sometimes, it may lead to a cytotoxic effect called autophagy‐dependent cell death.^15^ Therefore, autophagy has contrasting functions to protect or injure cells. There is currently no method to predict whether the induced autophagy will act cytoprotectively or cytotoxically.^15^

In NSCLC, high serum HSP70 levels are associated with an increased risk of developing,^16^ and the early stages of carcinogenesis are associated with high HSP70 expression.^17^ Furthermore, high HSP70 levels are related to the suppression of chemosensitivity and the effects of TKIs on prostate carcinoma, fibrosarcoma and chronic myeloid leukemia.^18^, ^19^, ^20^ Because HSP70 suppression in normal cells does not result in cell toxicity,^21^, ^22^ HSP70 inhibitors are expected to be novel therapeutic agents. Although the impact of HSP70 on tumor cells may depend on the cancer type, research on HSP70 in pleural mesothelioma remains inadequate. VER‐155008 is an adenosine‐derived HSP70 inhibitor,^23^ and we evaluated its antitumor effects via autophagy in pleural mesothelioma cell lines.

## Methods

### Cell lines and chemical reagents

The human pleural mesothelioma cell lines 211H, H2452 and H28 were cultured in RPMI 1640 medium (GE Healthcare, Japan, SH30027.01) supplemented with 10% fetal bovine serum (FBS) (Sigma‐Aldrich, USA) and 1% BIOMYC‐3 antibiotic solution (Biological Industries, USA, 03–038‐1B) at 37°C and 5% CO_2_. FBS was inactivated in 56°C water 30 minutes before use. VER‐155008 (R&D Systems, USA, 3803), cisplatin (FUJIFILM Wako Pure Chemical Corporation, Japan, 033–20091), gefitinib (Cayman Chemical, USA, 13166), or dimethylsulfoxide (DMSO) (FUJIFILM Wako Pure Chemical Corporation, Japan, 046–21 981) as a control was added to the cell culture media.

### Western blotting

Cells were lysed with the RIPA Lysis Buffer System (Santa Cruz Biotechnology, USA, sc‐24948). The cell lysates were then treated with a BCA protein assay kit (Thermo Fisher Scientific, USA, 23227), and absorbance was measured at a wavelength of 562 nm by an SH1300‐Lab instrument (Hitachi High‐Tech Science Corporation, Japan) with SF61 version 5.6.0 (CORONA ELECTRIC, Japan) (https://www.hitachi-hightech.com/hhs/products/mtp_sf6/) to determine the protein concentration. Cell lysates were mixed with 2x Laemmli sample buffer (Bio‐Rad, USA, 161–0737) containing 5% 2‐Mercaptoethanol (Bio‐Rad, USA, 161–0710). The aliquots (20 μg) were boiled at 95°C and separated on sodium dodecyl sulfate‐polyacrylamide gels (SDS‐PAGE; NextPage I, Gellex International, Japan, GLX‐1E4B) with an AccuRuler RGB Prestained Protein Ladder (Maestrogen, Taiwan, 02101–250) as molecular markers. The proteins separated by SDS‐PAGE were electrotransferred onto polyvinylidene difluoride membranes (Bio‐Rad, USA, 1620176) for protein blotting. After incubation with a 4% Block Ace powder (KAC, Japan, UK‐B80), 2% ECL Prime Blocking Reagent (Amersham, UK, RPN418), or EveryBlot Blocking Buffer (Bio‐Rad, USA, 12010020), the blots were incubated for 1 hour at room temperature with primary antibodies, which were diluted by 1000 with TBS‐T (20 mM Tris‐HCl, 150 mM NaCl, pH 7.4, and 0.05% TWEEN 20). After washing with TBS‐T, the blots were incubated for 1 hour at room temperature with secondary antibodies which were diluted by 50 000 with TBS‐T. Using ECL Prime western blotting detection reagent (Amersham, England, RPN2232), the antigen‐antibody complexes were detected by a ChemiDoc XRS+ System with Image Lab Software Version 2.0 build 8 or Version 6.0.1 build 34 Standard Edition (Bio‐Rad, USA) (https://www.bio-rad.com/en-us/product/image-lab-software). The primary antibodies were as follows: HSP70/HSC70 (W27) (sc‐24), p‐AKT1/2/3 (C‐11) (sc‐514032), GFP (B‐2) (sc‐9996) and β‐actin (C4) (sc‐47778) from Santa Cruz Biotechnology (USA); AKT (pan) (11E7) rabbit mAb (4685), and LC3A (D50G8) XP rabbit mAb (4599) from Cell Signaling Technology (USA); and anti‐LAMP2A (ab18528) from Abcam (UK). The secondary antibodies were anti‐mouse IgG, HRP‐linked whole Ab sheep (NA931) and anti‐rabbit IgG, HRP‐linked whole Ab donkey (NA934) from GE Healthcare (USA).

### Cell proliferation analysis

Mesothelioma cells were seeded onto 96‐well plates at 500 to 1000 cells per well and cultured overnight in RPMI 1640 containing 10% inactivated FBS without antibiotics. The medium was then changed to medium containing VER‐155008, cisplatin, gefitinib, or DMSO as a control. After culturing for 24, 48, or 72 hours at 37°C and 5% CO_2,_ 10.0 μL Cell Counting Kit‐8 reagent (Dojindo, Japan, CK04) was added to each well and cultured for two hours. Reduced water‐soluble tetrazolium salt‐8 was measured at a wavelength of 450 nm by an SH1300‐Lab instrument to evaluate cell viability.

### Colony formation

Mesothelioma cells were seeded onto six‐well plates at 1000 cells per well and cultured overnight in RPMI 1640 containing 10% inactivated FBS without antibiotics. The medium was then changed to medium containing 1.0–20.0 μM VER‐155008 or DMSO as a control. The cells were cultured for 10–14 days at 37°C and 5% CO_2_, and the colonies were stained with methylene blue for counting.

### Cell cycle analysis

Mesothelioma cells were seeded onto six‐well plates. After overnight incubation in RPMI 1640 containing 10% inactivated FBS without antibiotics, the medium was changed to medium containing 20.0 μM VER‐155008 or DMSO as a control. After culturing for a further 48 hours at 37°C and 5% CO_2,_ the cells were collected and stained with a BD Cycletest Plus DNA Reagent Kit (Becton, Dickinson Biosciences, USA, 340242), and the ratio for each cell cycle phase was measured by BD FACSVerse (Becton, Dickinson Biosciences, USA) with FACSuite v1.0.5.3841 (Becton, Dickinson Biosciences, USA) (https://www.bdbiosciences.com/en-us/instruments/research-instruments/research-software/flow-cytometry-acquisition/facsuite-software).

### Plasmid transfection

The pMRX‐IP‐GFP‐LC3‐RFP‐LC3ΔG plasmid was a gift from Professor Noboru Mizushima (Addgene, USA, plasmid #84572; http://n2t.net/addgene:84572; RRID: Addgene_84572).^24^ PLAT‐A cells, kindly gifted from Professor Toshio Kitamura, Division of Cellular Therapy, Institute of Medical Science, University of Tokyo, Tokyo, Japan, were cultured in Dulbecco's modified Eagle's medium (DMEM) with 4.5 g/L glucose (Sigma‐Aldrich, USA, D5796) and 10% heat inactivated FBS and seeded in 60 mm diameter dishes. A mixed solution of 200 μL Opti‐MEM (ThermoFisher Scientific, USA, 31985–070), 10 μg PEI‐MAX solution (Polysciences, Inc., USA, 24765–1) and 3 μg plasmid was then added to the media. After 48 hours of culture, the supernatant was collected as a virus stock. The virus stock with a 10.0 μg/mL polybrene solution (Nacalai Tesque, Japan, 12996–81) was added to target cells in 24‐well plates and cultured for 24 hours. These cells were cultured in RPMI1640 with 10% inactivated FBS and 1.0 μg/ml puromycin (Invitrogen, USA, ant‐pr‐1) to select infected cells. After puromycin selection, the cells were seeded at 1 cell per well in a 96‐well plate to remove the cells with homologous recombination. The cells that developed green and red colors were collected under a fluorescence microscope. These experiments were performed at a P2‐level laboratory and approved by the Recombinant DNA Experiment Safety Committee of Saitama Medical University (application number: 1391).

### Macroautophagy analysis

Mesothelioma cells, which were transfected with pMRX‐IP‐GFP‐LC3‐RFP‐LC3ΔG, were seeded onto six‐well plates and cultured overnight in RPMI 1640 containing 10% inactivated FBS without antibiotics. The medium was then changed to medium containing 20.0 μM VER‐155008, 5.0 μM gefitinib or DMSO as a control with or without 10% inactivated FBS. The medium without FBS was used to starve the cells. After culturing for 24 hours at 37°C and 5% CO_2_, the medium was removed, and the cells were fixed with methanol. The cells were then observed by a BZ‐X810 fluorescence microscope (Keyence, Japan) with BZ series application 01.01.00.17. Furthermore, the green fluorescence intensity of the collected cells was measured as fluorescein isothiocyanate (FITC), and the red fluorescence intensity was measured as phycoerythrin‐Cy5 A (PC5‐A) with a BD FACSVerse. The mean green and red fluorescence values were assessed, and macroautophagy activity was expressed as the ratio of the mean value of the green fluorescence to that of the red fluorescence.

### Statistical analysis

Analyses for two groups were performed with F tests and *t*‐tests. If the groups were assessed as having equal variance, a two‐sided *t*‐test was performed. If the groups were assessed as having unequal variance, two‐sided Welch's test was performed.

For multiple comparisons, Tukey's test was performed when we intended to compare all groups with each other. Dunnett's multiple comparison test was performed to compare each group with the control group.

Experiments were performed nine times for cell proliferation, and three times for colony formation, cell cycle and macroautophagy analysis, and these results were statistically analyzed.

Statistical analyses were performed using the EZR software program Version 2.5–1 (Saitama Medical Center, Jichi Medical University, Saitama, Japan)^25^ (https://socialsciences.mcmaster.ca/jfox/Misc/Rcmdr), and the level of significance was set at *P* < 0.05.

IC_50_ was calculated by drawing dose‐response curves using GraphPad Prism7 (GraphPad Software, Inc., USA) (https://www.graphpad.com).

Combination index (CI) was obtained by drawing combination index plots and normalized isobolograms based on the Chou‐Talalay method^26^ using CompuSyn version 1.0. (ComboSyn, Inc., USA) (http://www.combosyn.com).

## Results

### Effect of VER‐155008, an HSP70 inhibitor, on mesothelioma cell line proliferation

**Figure 1:**
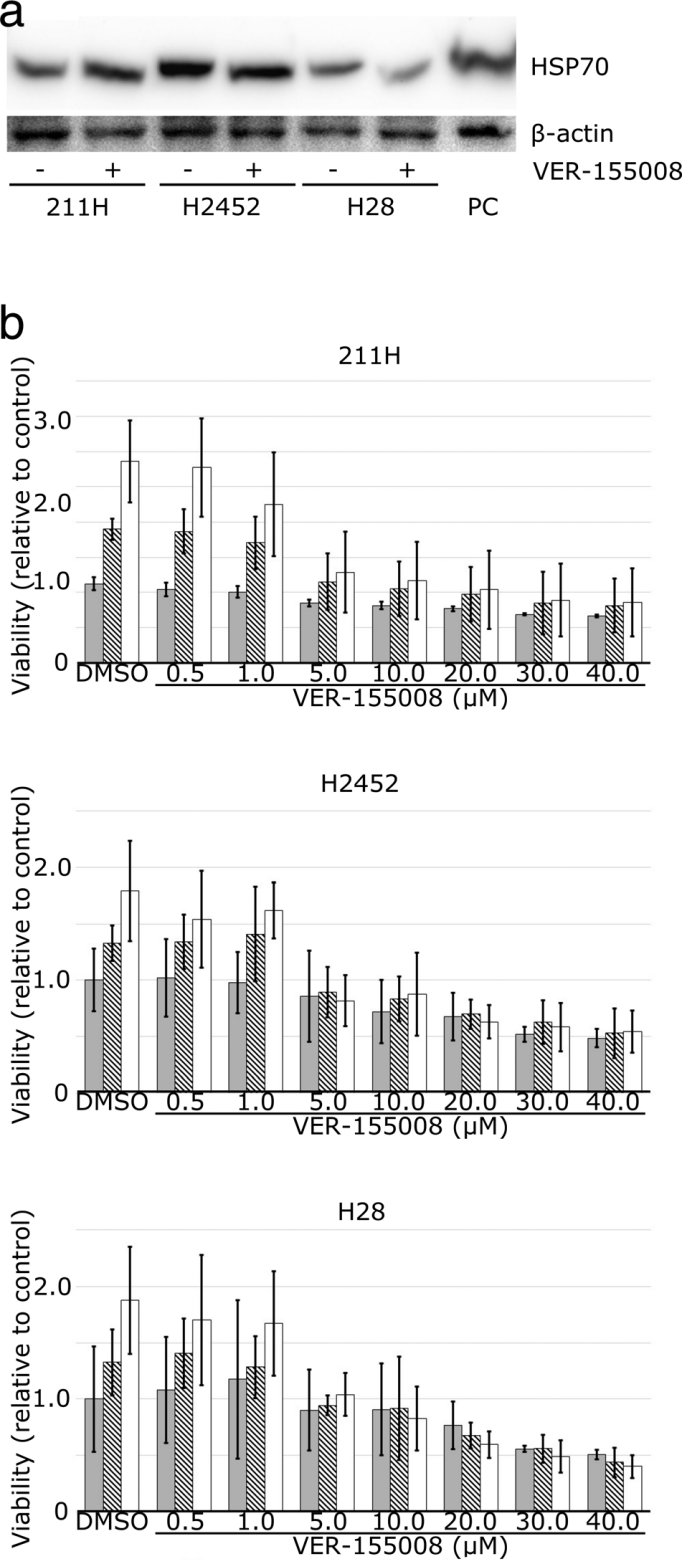
Heat shock protein 70 (HSP70) dysfunction induced by VER‐155008 suppressed mesothelioma cell proliferation. (**a**) Western blot analysis shows HSP70 expression in 211H, H2452 and H28 mesothelioma cells whose HSP70 expression was not affected by the addition of 20.0 μM VER‐155008. PC, positive control; β‐actin, internal control. (**b**) VER‐155008 suppressed the viability of 211H, H2452 and H28 mesothelioma cells. Cell viability was assessed by measuring the reduction of water‐soluble tetrazolium salt‐8. The results (ratio of viable cells treated with 0.5–40.0 μM VER‐155008 to those treated with dimethylsulfoxide [DMSO] for 24 hours as a control) at 24 hours (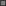), 48 hours (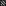) and 72 hours (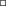) after the addition of VER‐155008 or DMSO are expressed as the mean ± SD (error bars).

The mesothelioma cell lines 211H, H2452 and H28 were cultured with 20.0 μM VER‐155008 for 72 hours, and protein was extracted. Western blot analysis showed that VER‐155008 did not alter HSP70 expression (Fig 1a). Indeed, VER‐155008 has been reported to suppress the function of HSP70 without reducing its expression.^27^

211H, H2452 and H28 cells were cultured for 72 hours with 0.5–40.0 μM VER‐155008. 211H cell viability was decreased by 5.0 μM VER‐155008 after 24 hours (*P*‐values for Tukey's test: 0.0000000 at 24 hours, 0.0012526 at 48 hours, and 0.0000119 at 72 hours), and H2452 and H28 cell viability was suppressed by 5.0 μM VER‐155008 after 48 hours (*P*‐values: 0.0057422 at 48 hours and 0.0000001 at 72 hours in H2452 cells, 0.0439949 at 48 hours and 0.0000601 at 72 hours in H28 cells) (Fig 1b). The viability of all cell lines was suppressed by 10.0–40.0 μM VER‐155008. The IC_50_ values at 72 hours after administration of VER‐155008 for 211H, H2452 and H28 were 2.2 μM, 1.5 μM and 3.1 μM, respectively.

**Figure 2:**
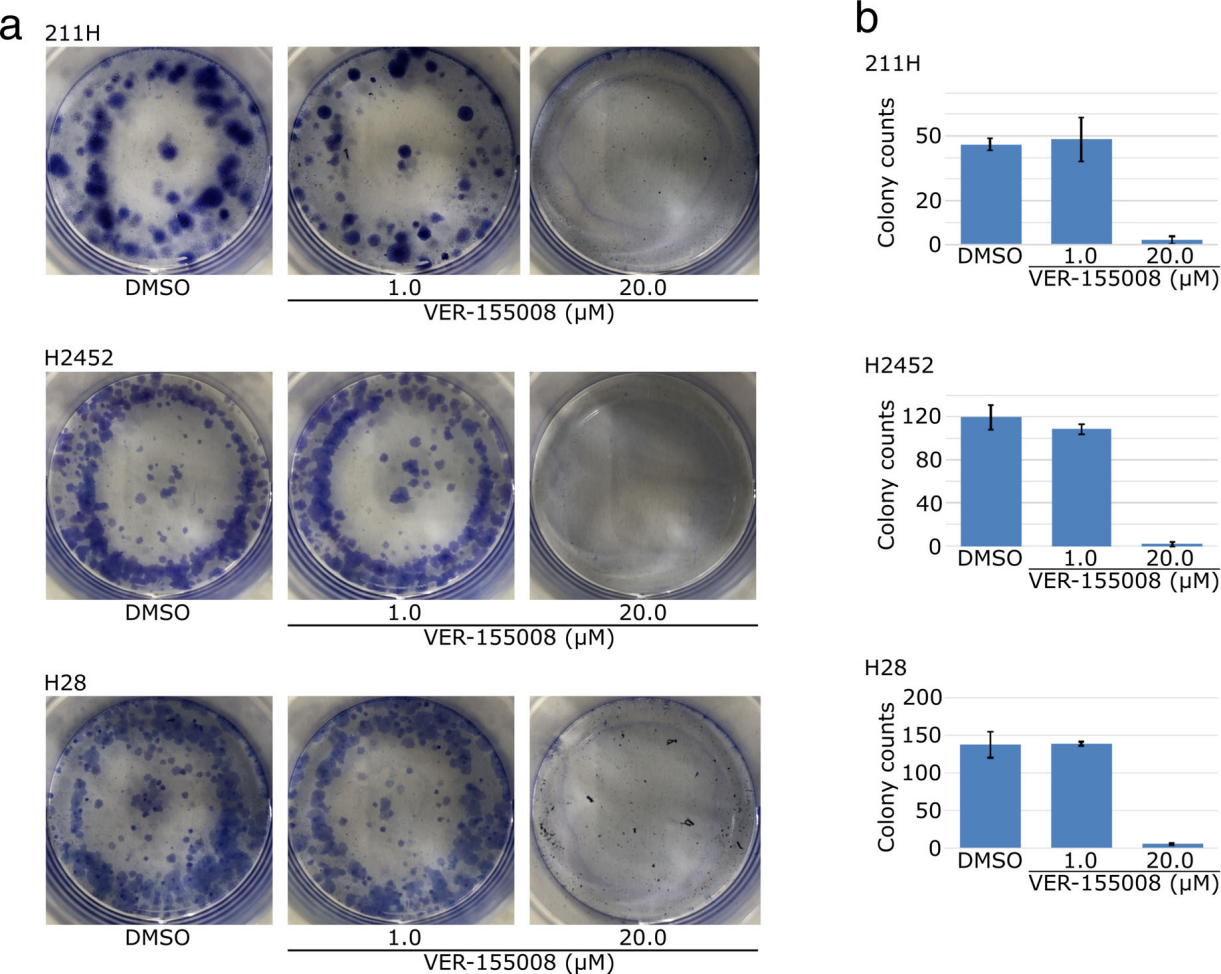
Heat shock protein 70 (HSP70) dysfunction induced by VER‐155008 suppressed colony formations of mesothelioma cells. (**a**) VER‐155008 suppressed the colony formation of 211H, H2452 and H28 mesothelioma cells. Cells were spread on dishes and cultured between 10 and 14 days with 1.0–20 μM VER‐155008 or dimethylsulfoxide (DMSO). (**b**) The colonies formed were counted and are expressed in bar graph as the mean ± SD (error bars).

When the colony formation effects of VER‐155008 were evaluated, 20.0 μM VER‐155008 was shown to inhibit colony formation in 211H, H2452 and H28 cells (Fig 2).

**Figure 3:**
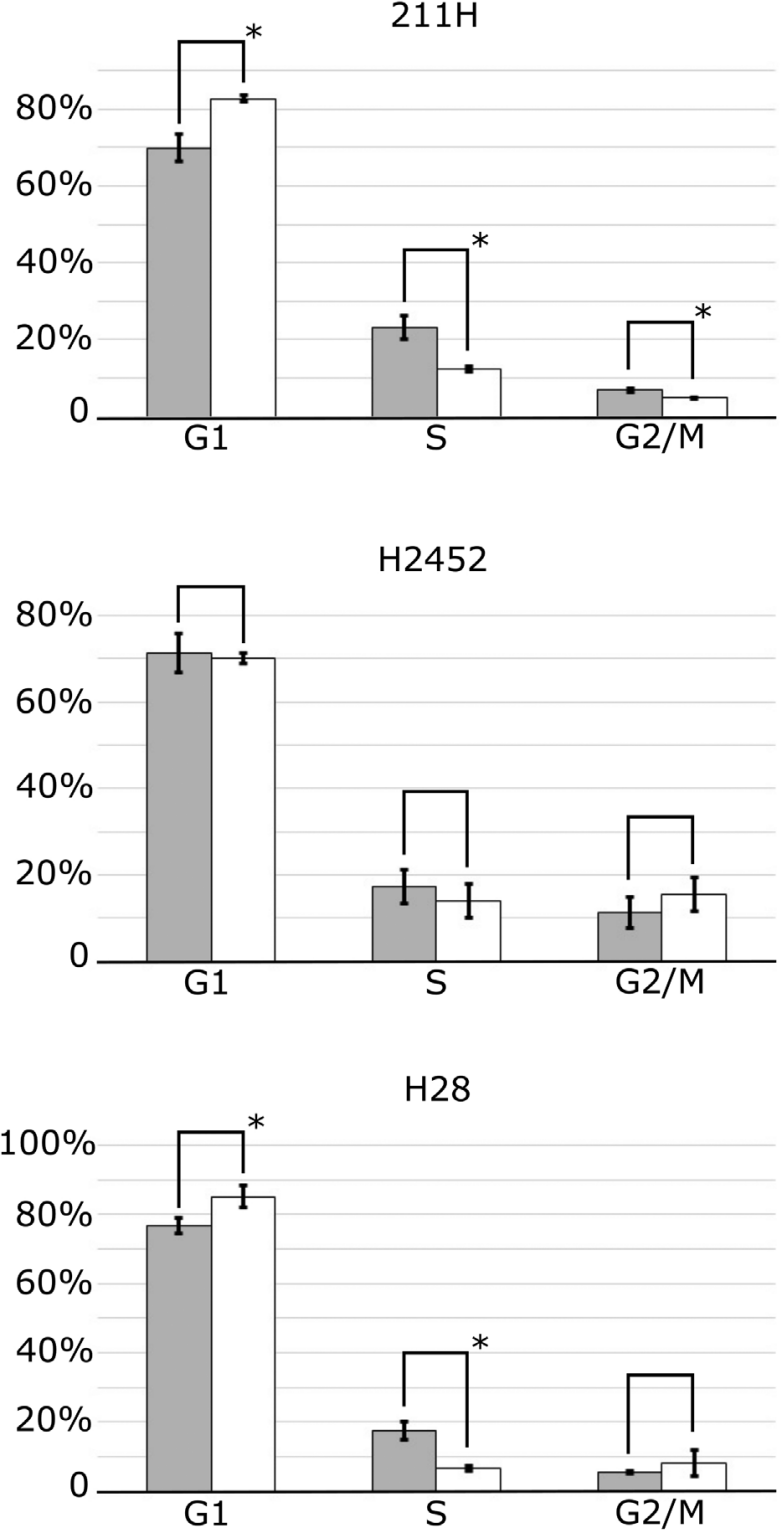
Heat shock protein 70 (HSP70) dysfunction by VER‐155008 induced cell cycle aberration in mesothelioma cells. The proportion of G_1_, S or G_2_/M phase is expressed as the mean ± SD (error bars). The proportion of G_1_ phase 211H and H28 cells was higher after culture with 20.0 μM VER‐155008 for 48 hours, compared to those with control dimethylsulfoxide (DMSO). *P < 0.05. (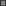) DMSO, (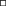) 20 μM VER‐155008.

The G_1_ phase cell population was increased after 48 hours of culture with 20.0 μM VER‐155008 compared to culture with DMSO for both 211H and H28 cells (82.7 ± 0.9% for VER‐155008 to 69.9 ± 3.5% for DMSO, *P* = 0.003871, in 211H cells; 85.2 ± 3.3% for VER‐155008 to 77.0 ± 2.3% for DMSO, *P* = 0.008483, in H28 cells) (Fig 3). However, the G_1_ phase cell population was not altered by VER‐155008 in H2452 cells (70.0 ± 1.2% for VER‐155008 to 71.3 ± 4.6% for DMSO).

### Effect of VER‐155008 in combination with cisplatin or gefitinib on mesothelioma cell proliferation

**Figure 4:**
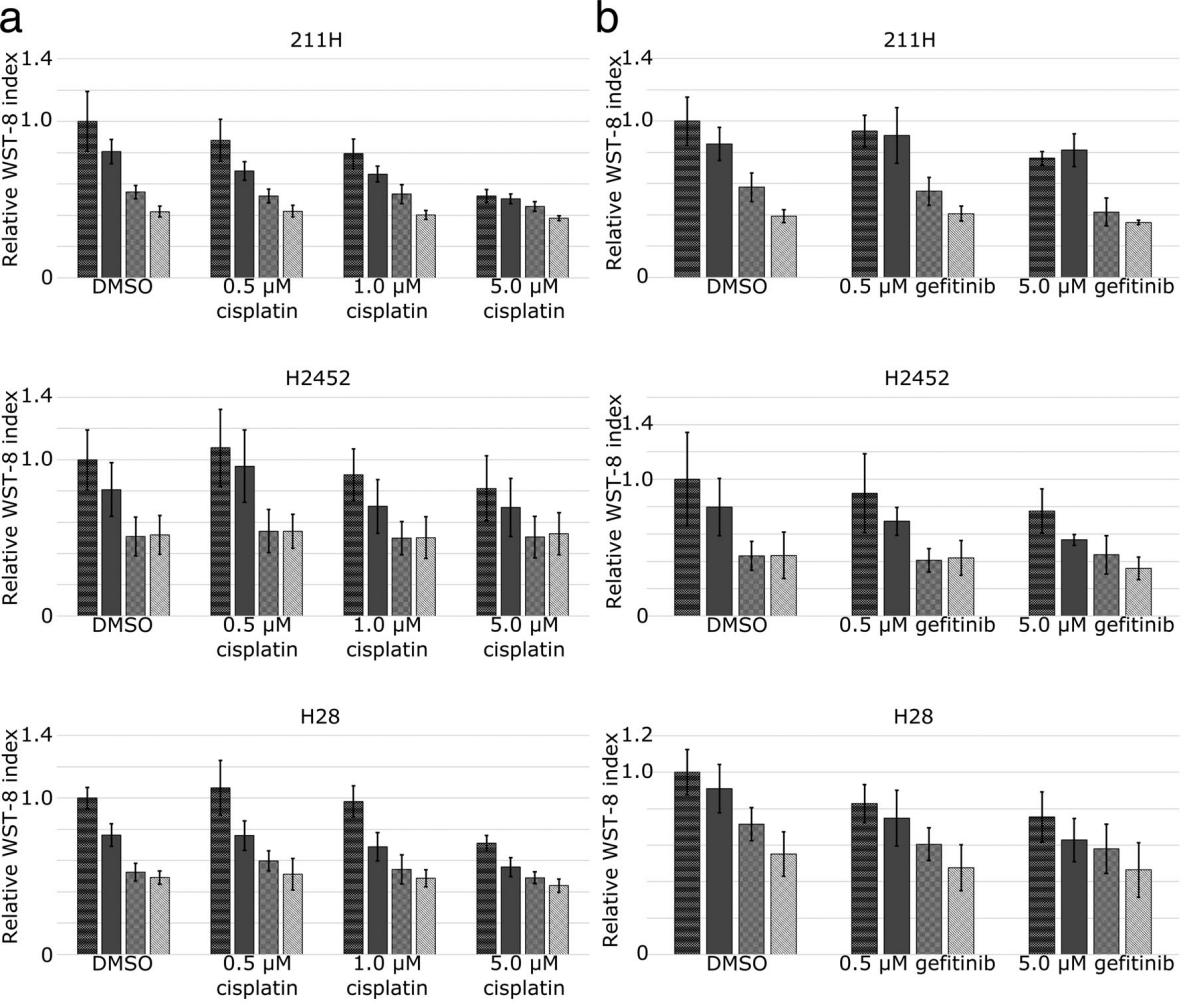
Mesothelioma cell growth suppression by VER‐155008 was not enhanced by the addition of cisplatin (**a**) or gefitinib (b). (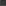 ) DMSO, (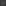 ) 1.0 μM VER‐155008, (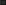 ) 5.0 μM VER‐155008, (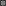 ) 20.0 μM VER‐155008. Mesothelioma cells, 211H, H2452 and H28, were cultured for 48 hours with 0.5–5.0 μM cisplatin, 0.5–5.0 μM gefitinib, or dimethylsulfoxide (DMSO) as a control, in addition to VER‐155008 at concentrations of 1.0 μM, 5.0 μM and 20.0 μM or DMSO. Cell viability was assessed by the reduction of water‐soluble tetrazolium salt‐8. The results (ratio of viable cells treated with cisplatin or gefitinib combined with VER‐155008 to those treated with DMSO for 48 hours) are expressed as the mean ± SD (error bars).

Cisplatin has the ability to suppress mesothelioma cell growth and is used with pemetrexed as a key drug for mesothelioma treatment.^28^ When 211H, H2452 and H28 cells were cultured for 48 hours with the combination of 0.5–5.0 μM cisplatin and 1.0–20.0 μM VER‐155008, cell viability was measured. Both cisplatin and VER‐155008 suppressed tumor cell growth, yet the combination of cisplatin and VER‐155008 did not enhance the cell‐suppressing effect (Fig 4a).

Although mesothelioma cells are known to overexpress EGFR, clinical trials of EGFR inhibitors for pleural mesothelioma have not proven their effectiveness.^29^ Therefore, the antitumor effect of gefitinib against pleural mesothelioma upon HSP70 inhibition was investigated. 211H, H2452 and H28 cells were cultured for 48 hours in combination with 0.5–5.0 μM gefitinib and 1.0–20.0 μM VER‐155008. Gefitinib did not adequately suppress mesothelioma cells growth, and the addition of VER‐155008 did not enhance the cell growth inhibition in 211H and H2452, while H28 tended to be synergistically suppressed as the CI by the Chou‐Talalay method^26^ (Fig 4b).

### Effect of VER‐155008 on HSP70‐related proteins in mesothelioma cells

**Figure 5:**
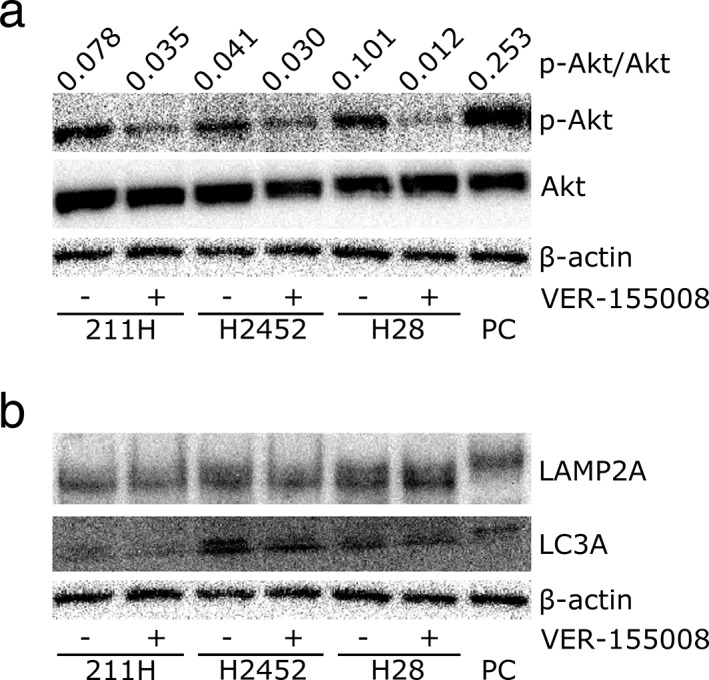
VER‐155008 inhibited heat shock protein 70 related protein expression. (**a**) Western blot analysis shows that VER‐155008 treatment resulted in downregulation of phosphorylated (p)‐AKT, while total AKT was stable after VER‐155008 addition. PC, positive control; β‐actin, internal control. (**b**) Western blot analysis showed no alteration on the expression of macroautophagy‐related marker, light chain 3A (LC3A) or chaperone‐mediated autophagy‐related markers, lysosome‐associated membrane protein 2A (LAMP2A). PC, positive control; β‐actin, internal control.

We investigated how HSP70 inhibition by VER‐155008 functions in mesothelioma cell proliferation. AKT is a candidate for association with HSP70 in cell proliferation signaling pathways, such as Wnt^30^ and EGFR.^11^ In 211H, H2452 and H28 mesothelioma cells, phosphorylated (p‐)AKT was decreased after culture with VER‐155008, while the protein expression of total AKT was not altered (Fig 5a). p‐AKT is expected to be involved in macroautophagy by activating mTOR, which negatively regulates macroautophagy.^31^ Signals from insulin that fluctuate depending on nutritional status have been reported to regulate macroautophagy in a suppressive manner via PI3K–AKT–FOXO signals.^32^ Our western blot results and previous findings suggested that HSP70 is associated with phosphorylation of AKT in this pathway to suppress macroautophagy. Next, we examined alterations in autophagy‐related proteins due to VER‐155008 by western blotting. Expression of light chain 3A (LC3A), which lines autophagosomes,^33^ was not changed by VER‐155008 (Fig 5b). Neither was the expression of LAMP‐2A, through which degraded proteins recognized by Hsc70, a cofactor of HSP70, are translocated to lysosomes in CMA.^34^ Since western blot analyses of these proteins might not be adequate to estimate autophagy, we analyzed macroautophagy using a plasmid system.

### **Effect of** **HSP70** **inhibition by** **VER** **‐155008 on autophagy**

211H, H2452 and H28 cells were transfected with the pMRX‐IP‐GFP‐LC3‐RFP‐LC3ΔG plasmid^24^ by retrovirus infection. Transfected cells produce GFP‐LC3‐RFP‐LC3ΔG, and ATG4 separates it into GFP‐LC3 and RFP‐LC3ΔG. GFP‐LC3 outlines autophagosomes and is degraded by lysosomes when macroautophagy occurs. Therefore, green fluorescence is decreased as macroautophagy is activated; on the other hand, the red fluorescence of RFP‐LC3ΔG acts as an internal control. When the ratio of the green fluorescence signal to the red fluorescence signal (GFP/RFP) decreases, macroautophagy is activated.

**Figure 6:**
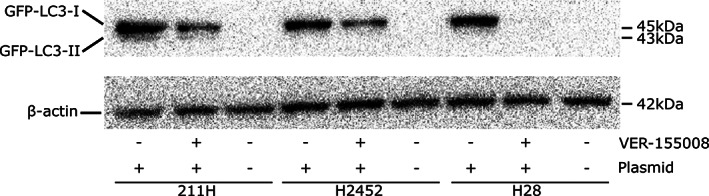
A plasmid system (pMRX‐IP‐GFP‐LC3‐RFP‐LC3ΔG) was transfected to mesothelioma cells for detection of macroautophagy. Western blots using a green fluorescent protein (GFP) antibody confirmed light chain 3 (LC3) expression in mesothelioma cells after plasmid introduction compared to cells without the plasmid. β‐actin, internal control.

Transfected cells were cultured for 24 hours with or without 20 μM VER‐155008 and collected for protein extraction. Western blot analysis revealed GFP‐LC3‐I and GFP‐LC3‐II expression in transfected cells (Fig 6). In contrast, this expression did not appear in nontransfected cells. The GFP‐LC3‐I and GFP‐LC3‐II expression level to β‐actin expression as an internal control in 211H cultured without or with VER‐155008 was 1.48 and 0.11, and 0.55 and 0.02, respectively. In H2452, the GFP‐LC3‐I expression level without or with VER‐155008 was 0.78 and 0.37, and GFP‐LC3‐II was not detected. In H28, the GFP‐LC3‐I expression level without VER‐155008 was 0.77, while GFP‐LC3‐I was not detected with VER‐155008, and GFP‐LC3‐II was not detected without or with VER‐155008.

**Figure 7:**
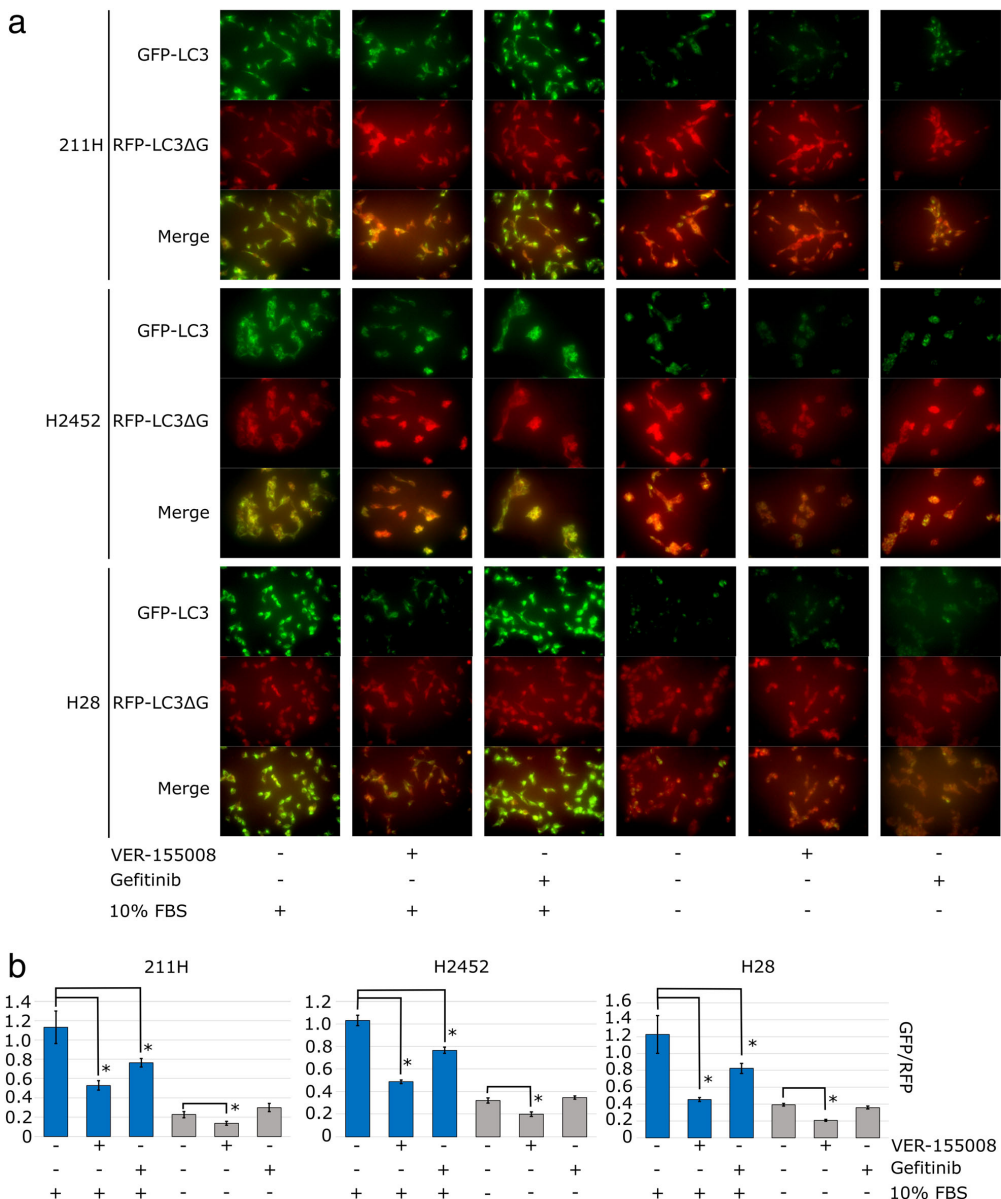
The effects of VER‐155008, gefitinib or FBS deprivation on macroautophagy in mesothelioma cells were assessed by a plasmid system (pMRX‐IP‐GFP‐LC3‐RFP‐LC3ΔG). This plasmid produces light chain 3 (LC3) protein bound to green fluorescent protein (GFP), and LC3ΔG protein, an internal control, bound to red fluorescent protein (RFP). (**a**) Fluorescent microscopy findings for transfected mesothelioma cells cultured for 24 hours with dimethylsulfoxide (DMSO), 20 μM VER‐155008 or 5.0 μM gefitinib under 10% fetal bovine serum (FBS) or FBS deprivation conditions. Scale bars = 100 μM. (**b**) The signal intensities of GFP and RFP were measured by flow cytometry after culture for 24 hours with DMSO, 20.0 μM VER‐155008 or 5.0 μM gefitinib under 10% FBS (blue bars) or FBS deprivation conditions (gray bars). The GFP/RFP signal intensity is expressed as the mean ± SD (error bars). *P < 0.05.

Macroautophagy in mesothelioma cells was evaluated by fluorescence microscopy (Fig 7a). 211H, H2452 and H28 cells were cultured for 24 hours with 20.0 μM VER‐155008 or 5.0 μM gefitinib with 10% inactivated FBS (FBS‐supplemented condition) or without FBS (FBS‐deprived condition). Mesothelioma cells under FBS‐supplemented conditions showed stronger GFP‐LC3 emission than RFP‐LC3ΔG emission. VER‐155008 addition to this condition decreased GFP emission, while gefitinib addition slightly lessened it. On the other hand, mesothelioma cells under FBS‐deprived conditions clearly showed a decrease in the GFP signal; therefore, the merged images appear red. From these results, fluorescence microscopy showed that in 211H, H2452 and H28 cells, GFP‐LC3 was decreased by VER‐155008 and FBS‐deprived conditions, indicating macroautophagy activation.

Next, the GFP and RFP emission intensity was quantitatively measured by flow cytometry (Fig 7b). In 211H, H2452 and H28 cells under FBS‐supplemented conditions, flow cytometry indicated that GFP/RFP was significantly decreased by the addition of VER‐155008 (Dunnett's test: *P* = 0.000761, 0.00000177 and 0.000746 in 211H, H2452 and H28 cells, respectively) and gefitinib (Dunnett's test: *P* = 0.009209, 0.000115 and 0.018267 in 211H, H2452 and H28 cells, respectively) compared to medium containing DMSO as a control. Under FBS‐deprived conditions, the GFP/RFP ratio was decreased by adding VER‐155008 (Dunnett's test, *P* = 0.0241, 0.000494 and 0.0000102 in 211H, H2452 and H28 cells, respectively) but not by adding gefitinib (Dunnett's test, *P* = 0.0581, 0.248053 and 0.0604 in 211H, H2452 and H28 cells, respectively) compared to adding DMSO as a control. From the quantification of these flow cytometry results, VER‐155008 caused macroautophagy in FBS‐supplemented conditions, and this effect was significantly enhanced in FBS‐deprived conditions; however, gefitinib activated macroautophagy in only FBS‐supplemented conditions.

**Figure 8:**
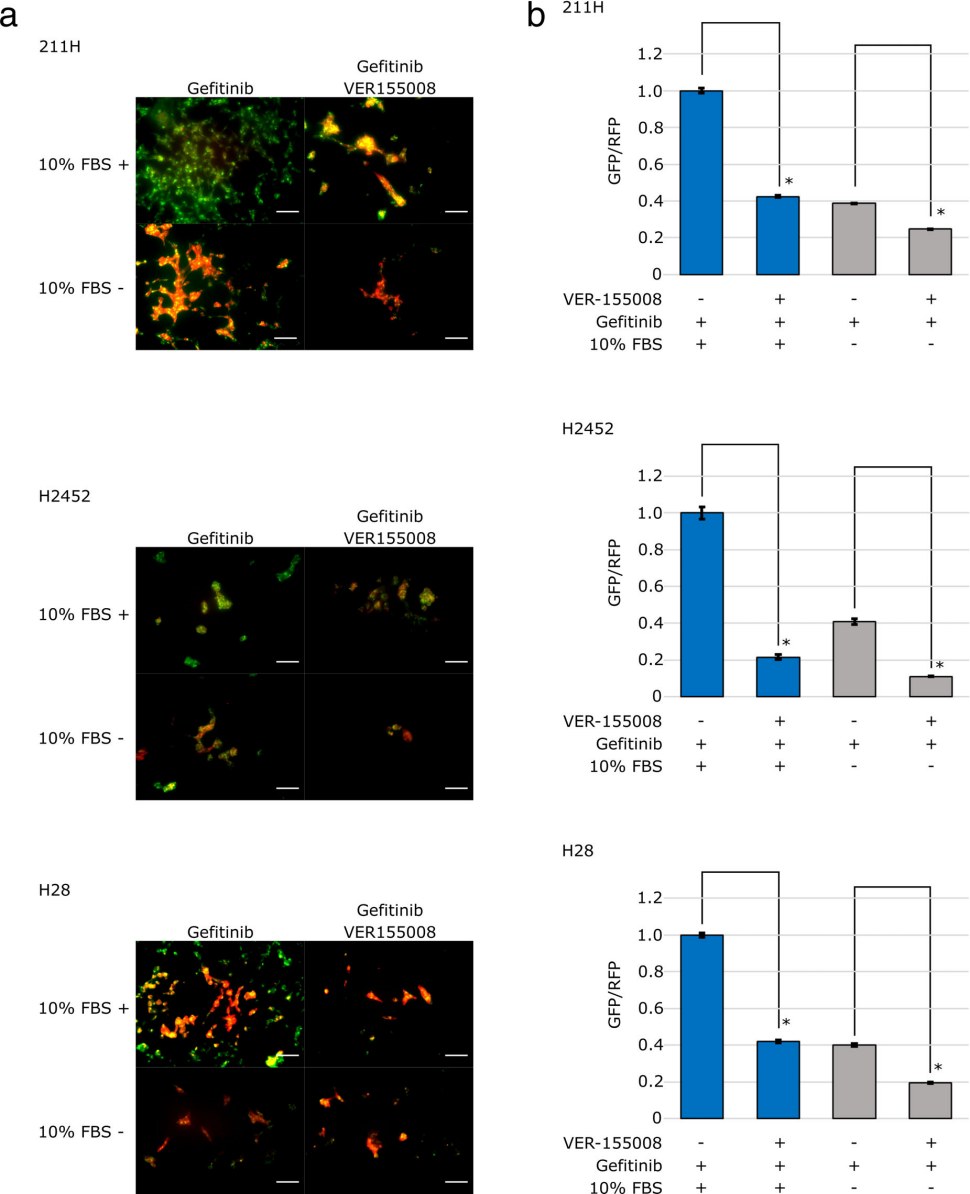
The combination of VER‐155008 and gefitinib enhanced the macroautophagy with gefitinib alone under the fetal bovine serum (FBS) supplemented or the FBS‐deprived conditions. (**a**) Fluorescent microscopy findings for transfected mesothelioma cells cultured for 24 hours with 5.0 μM gefitinib or the combination of 20 μM VER‐155008 and 5.0 μM gefitinib under 10% FBS or FBS deprivation conditions. Scale bars = 100 μm. (**b**) The signal intensities of GFP and RFP were measured by flow cytometry after culture for 24 hours with 5.0 μM gefitinib or the combination of 20 μM VER‐155008 and 5.0 μM gefitinib under 10% FBS (blue bars) or FBS deprivation conditions (gray bars). The GFP/RFP signal intensity is expressed as the mean ± SD (error bars). *P < 0.05.

Furthermore, we confirmed whether the combination of VER‐155008 and gefitinib enhanced the macroautophagy with gefitinib alone in the FBS‐supplemented or FBS‐deprived conditions. Fluorescence microscopy findings showed further reduction of GFP‐LC3 by the addition of 20.0 μM VER‐155008 and 5.0 μM gefitinib compared to the addition of 5.0 μM gefitinib alone in 211H, H2452 and H28 cells cultured for 24 hours in FBS‐supplemented or FBS‐deprived conditions (Fig 8a). Flow cytometry quantification indicated that the combination of VER‐155008 and gefitinib significantly decreased the GFP/RFP intensity compared to the medium containing gefitinib alone in FBS‐supplemented condition (*t*‐test: *P* = 0.000000291, 0.00000292 and 0.000000255 in 211H, H2452 and H28 cells, respectively), and in FBS‐deprived conditions (*t*‐test: *P* = 0.000000266, 0.00000694 and 0.00000207 in 211H, H2452 and H28 cells, respectively) (Fig 8b).

## Discussion

From the results of this research, functional HSP70 inhibition by VER‐155008 treatment (5.0 μM or more) decreased mesothelioma cell viability in 221H, H28 and H2452 cell lines, and G_1_ arrest was induced in 211H and H28 cells, but not in H2452 cells. HSP70 inactivation by VER‐155008 inactivated AKT through phosphorylation; thus, downstream of PI3K might be inhibited, leading to G_1_ arrest and proliferation inhibition in mesothelioma cells. VER‐155008 has previously been reported to decrease the viability of lung cancer cells,^27^ in which PI3K inhibition leads to G_1_ arrest.^6^ These results suggested that HSP70 may regulate the pathway including PI3K/AKT/mTOR by a mechanism that differs from its well known function in regulating protein refolding as a molecular chaperone. In H2452, cell viability was suppressed by VER‐155008 without G_1_ arrest induction, which means that other processes, such as autophagy, than the above‐mentioned mechanism may be involved in cell proliferation.

Cisplatin has recently become a key drug for mesothelioma treatment, and the combination of cisplatin and HSP70 inhibition increased apoptosis in cervical squamous cell carcinoma and gastric cancer.^35^, ^36^ Although an additive antitumor effect was expected, this did not occur when VER‐155008 was combined with cisplatin in mesothelioma cell lines. In cervical squamous cell carcinoma, HSP70 knockdown by small interfering RNA (siRNA) enhanced cisplatin‐induced apoptosis.^36^ In gastric cancer, HSP70 inhibition by pifithirin‐μ, also known as a p53 inhibitor, or short hairpin RNA (shRNA) also enhanced cisplatin‐induced apoptosis.^35^ In this study, phosphorylation of p38, ERK and JNK was facilitated following cisplatin treatment, but AKT was not affected, and HSP70 knockdown inhibited phosphorylation leading to augmentation of apoptosis. VER‐155008 might work differently on cisplatin‐induced effects from HSP70 siRNA or shRNA, which suppresses the protein expression, and pifithirin‐μ, which inhibits p53 function in addition to HSP70 inhibition. Furthermore, cisplatin‐induced effects or the cooperative function of HSP70 inhibition and cisplatin might be different depending on cancer cell types. Gefitinib did not show an antitumor effect, even in mesothelioma cell lines with high EGFR expression,^29^ and the combination of VER‐155008 and gefitinib had no additive antitumor effect. Among signaling pathways, gefitinib and HSP70 dysfunction can both inhibit the PI3K pathway; however, gefitinib and HSP70 dysfunction do not exhibit a cooperative effect on cell proliferation. From our results, VER‐155008 in combination with cisplatin or gefitinib did not show additive antitumor effects over VER‐155008 alone.

In the present study, we used the novel plasmid pMRX‐IP‐GFP‐LC3‐RFP‐LC3ΔG to quantitatively and statistically evaluate macroautophagy, which cannot be monitored using the conventional method of detecting LC3‐I to LC3‐II conversion. While augmentation in LC3‐II expression on the western blot is regarded to imply the progression of autophagy, it can also represent a block in later stages of the autophagy pathway.^37^ Therefore, the conventional western blot analysis is inadequate to measure autophagy progression. The novel plasmid system enabled us to observe the complete autophagy pathway including the later stages. VER‐155008 induced the decrease both in the GFP‐LC3‐I and GFP‐LC3‐II, suggesting the progression of autophagy including the later stage, which was supported by the decrease in GFP/RFP in the plasmid pMRX‐IP‐GFP‐LC3‐RFP‐LC3ΔG system. In this research, gefitinib quantitatively induced macroautophagy in mesothelioma cells; however, under FBS‐deprived conditions, gefitinib did not quantitatively augment macroautophagy. The PI3K pathway, which is downstream of EGFR, is inhibited by gefitinib, and it is also regulated by increases or decreases in serum glucose levels. Our results suggested that the effects of gefitinib and FBS‐deprived conditions on the PI3K pathway might not cooperatively contribute to macroautophagy. On the other hand, HSP70 inhibition by VER‐155008 induced macroautophagy, which was additively enhanced under FBS‐deprived conditions. The combination of VER‐155008 and gefitinib enhanced the macroautophagy with gefitinib alone under FBS‐supplemented or FBS‐deprived conditions, which suggests that the mechanism of macroautophagy induction by HSP70 inhibition is related to other than the downstream of EGFR. Macroautophagy was originally known to protect cells because it is induced when the accumulation of destructive three‐dimensional protein structures exceeds the HSP70 refolding capacity.^8^ However, the effects of macroautophagy on cell survival have not been fully elucidated and remain debatable. In our study, the proliferation of mesothelioma cell lines was suppressed, accompanied by significant macroautophagy induction. We assumed two functions of macroautophagy in mesothelioma cells under HSP70 inhibition. One is the cell‐protective function of macroautophagy, and the other is autophagy‐dependent cell death to eliminate irreversibly damaged cells.^38^ So far, it has not been possible to identify either. HSP70 is also the only chaperone that controls CMA. Although HSP70 inhibition has been shown to suppress CMA, LAMP‐2A, a surrogate marker of CMA, did not change upon HSP70 inhibition in mesothelioma cells. Since this result may be attributed to the sensitivity of the detection methods, a novel measurement system for CMA is necessary.

Our study revealed that inhibiting HSP70 by VER‐155008 disrupts the PI3K/AKT/mTOR pathway to suppress mesothelioma cell line proliferation by inducing G_1_ cell cycle arrest, in addition to inhibiting protein refolding which is a known function of HSP70. Quantitative analysis of macroautophagy, such as via the method using the pMRX‐IP‐GFP‐LC3‐RFP‐LC3ΔG plasmid, enables the comparison of the effects between different cells or administered reagents. This study showed the difference in autophagy induction under FBS‐deprived conditions between VER‐155008 and gefitinib treatment. While gefitinib did not show synergistic macroautophagy induction under FBS‐deprived conditions, VER‐155008 administration under FBS‐deprived conditions resulted in synergistic macroautophagy induction, indicating that other autophagy‐related mechanisms are affected by functional HSP70 suppression by VER‐155008 rather than the PI3K/AKT/mTOR pathway, which can be suppressed by gefitinib. Although whether macroautophagy works to protect or injure cells needs further consideration, quantitative observation of macroautophagy by this plasmid system with an internal control will further be needed for situations with both cell proliferation suppression and macroautophagy induction, such as during HSP70 inhibition. These observations which clarify the function of macroautophagy are helpful for developing a new strategy for devastating malignancies such as mesothelioma.

## Disclosure

Kosuke Sakai received research funding from Eli Lilly Japan K.K. Masahiro Seike received research funding from Chugai Pharmaceutical Co. and TAIHO Pharmaceutical Co. and honoraria from AstraZeneca K.K., Chugai Pharmaceutical Co. and MSD K.K. Akihiko Gemma received an honoraria from AstraZeneka Co. Kazutsugu Uematsu received research funding from Chugai Pharmaceutical Co., Nippon Boehringer Ingelheim Co., Novartis Pharma K.K. and TAIHO Pharmaceutical Co. The rest of the authors have no potential conflicts of interest.
